# Ocular surface mucins and local inflammation—studies in genetically modified mouse lines

**DOI:** 10.1186/s12886-015-0137-5

**Published:** 2015-12-17

**Authors:** Kumi Shirai, Shizuya Saika

**Affiliations:** Department of Ophthalmology, Wakayama Medical University School of Medicine, 811-1 Kimiidera, Wakayama, 641-0012 Japan

**Keywords:** Mucin, Cornea, Conjunctiva, Epithelium, Inflammation, Mouse

## Abstract

Mucins locate to the apical surfaces of all wet-surfaced epithelia including ocular surface. The functions of the mucins include anti-adhesive, lubrication, water retention, allergens and pathogen barrier function. Ocular surface pathologies, i.e. dry eye syndrome or allergic conjunctivitis, are reportedly associated with alteration of expression pattern of mucin components. Recent investigations indicated anti-bacterial adhesion or anti-inflammatory effects of members of mucins in non-ocular tissues, i.e., gastrointestinal tracts or airway tissues, by using genetically modified mouse lines that lacks an expression of a mucin member. However, examination of ocular phenotypes of each of mucin gene-ablated mouse lines has not yet fully performed. Muc16-dficient mouse is associated with spontaneous subclinical inflammation in conjunctiva. The article reviews the roles of mucin members in modulation of local inflammation in mucous membrane tissues and phenotype of mouse lines with the loss of a mucin gene. Analysis of ocular surface of mucin-gene related mutant mouse lines are to be further performed.

## Introduction

Mucins are a class of high-molecular weight hydrophilic glycoproteins that contain multiple tandem repeats of amino acids that are rich in serine and threonine in the central domain of the core peptide [1] and locate to the apical surfaces of all wet-surfaced epithelia [2]. The number of amino acids per tandem repeat differs among each mucin molecule [3, 4]. Epithelial mucins are categorized as secreted and membrane-associated mucins (MAMs). Secreted mucins have no transmembrane-spanning domains and are produced by goblet cells [5]. The secreted mucins have the capability to capture allergens, cell debris and pathogens to facilitate their clearance from mucosal surfaces [6]. MAMs have a short cytoplasmic tail, a single transmembrane domain, an autoproteolytic domain, and a large, heavily glycosylated extracellular domain that contributes to the formation of the glycocalyx of apical cells in wet-surfaced epithelia [2]. Although the functions of the MAMs include anti-adhesive, lubrication, water retention, pathogen barrier function [2, 7], Recent investigations indicate anti-inflammatory effects of mucins in gastrointestinal tract or eye conjunctiva [8, 9]. In the current article the importance of mucins in ophthalmological clinical setting was described. Although cell culture studies on the roles of mucins have intensively conducted, they failed to figure out the roles of each mucin members in vivo. Such disadvantage of in vitro studies has well overcome by the availability of genetically mucin-gene-modified mouse lines. The roles of mucins in maintenance of ocular surface homeostasis as revealed by using mouse lines with ablation of each mucin gene were reviewed with a special interest in the regulation of tissue inflammation in the ocular surface inflammation.

### Classification of mucins and their distribution in the ocular surface

**Fig. 1:**
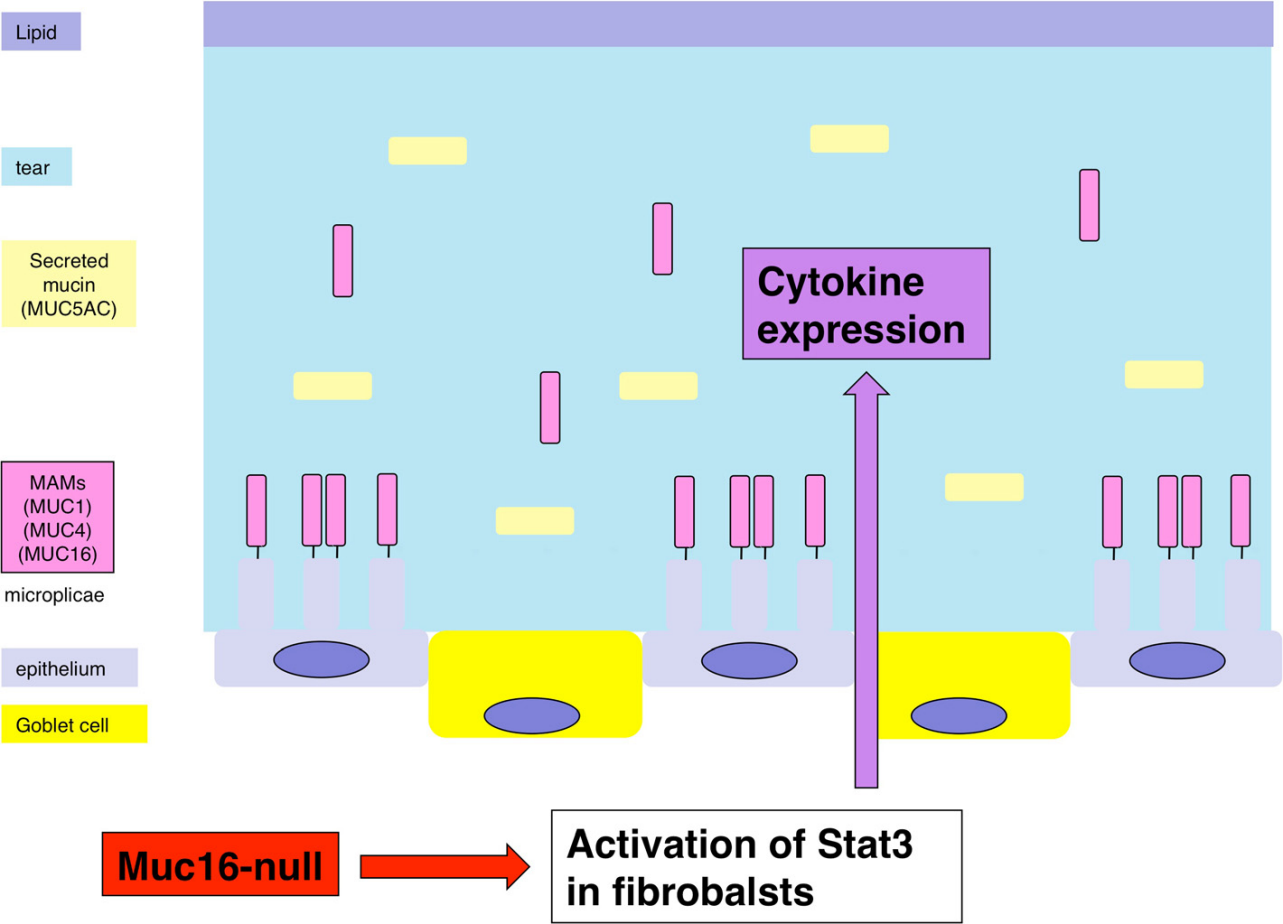
Ocular surface mucins. The ocular surface epithelia express three MAMs, that are concentrated on the tips of the apical cells’ microplicae. Secreted mucins are produced by Goblet cells and secreted in the tear film. The loss of Muc16 activates Stat3 signaling in fibroblasts in the subconjunctival connective tissue and upregulates expression of cytokine. Reproduced from [67]

Mucins identified in humans are designated as MUC, mouse homologues are identified by Muc and rat homologues are identified by rMuc. Epithelial mucins are categorized as secreted mucins and MAMs (Table 1). Secreted mucins including MUC2 and MUC5AC (Muc5AC in mice) have no transmembrane-spanning domains and are produced by goblet cells [5]. MUC5AC tend to form polymers in the goblet cells, where they are stored, but are secreted as monomers in the tear film (Fig. 1) [10]. MUC5AC spreads in tear film and decreases in density from the epithelial surface toward the lipid layer [11]. MUC5AC reportedly has the capability to trap allergens, debris and pathogens in order to facilitate their clearance from mucosal surfaces [6].

**Table 1:** Characteristic of ocular surface mucins

	Type	Chromosome position
MUC1	membrane asociated	1q21-q23
MUC4	membrane asociated	3q29
MUC5AC	secretory	11p15
MUC16	membrane asociated	19p13 · 2

MAMs, i.e., MUC1, MUC4 and MUC16 (Muc1, Muc4 and Muc16 in mice), have a short cytoplasmic tail, a single transmembrane domain, an autoproteolytic domain, and a large, heavily glycosylated extracellular domain that contributes to the formation of the glycocalyx of apical cells in wet-surfaced epithelia [2]. The ocular surface epithelia express three MAMs (MUC1, MUC4 and MUC16), that are concentrated on the tips of the apical cells’ microplicae, forming a dense glycocalyx at the epithelial-tear film interface [2], but MAM’s extracellular domains can also be released from the epithelial cell surface as soluble forms and are found in the tear film (Shedding) (Fig. 1) [10, 12]. MUC1 is expressed in both corneal and conjunctival epithelia [13, 14]. Interestingly the cytoplasmic domain of MUC1 play a role similar to transcription factor when being freed from cytoplasmic membrane; cytoplasmic tail of MUC1 interacts with β-catenin, is transported to the nucleus and modulates transcription of genes involved in cell proliferation and differentiation [15, 16]. MUC4 is most prevalent in conjunctival epithelia with an apparent diminution toward the central corneal epithelium in humans [14, 17]. In contrast, there is a high level of expression of Muc4 and rMuc4in the conjunctiva and cornea of mice and rats [18, 19]. MUC16 is initially known as the CA125 tumor antigen, that is commonly used as a marker of the presence and progression of certain malignant tumors [20–22]. MUC16/CA125 was then found to be widely expressed in normal tissues as a member of MAMs. In the ocular tissue MUC16 is expressed in epithelia of both cornea and conjunctiva in human [14, 23], but only in conjunctiva in mice [9, 24]. MUC16 is localized in the microplicae on the surface of the corneal and conjunctival epithelia, and bind to the actin cytoskeleton with its cytoplasmic tails [25], suggesting the roles of Muc16 in the maintenance of the microplicae architecture.

### The roles of mucins in ocular surface

The secreted mucins have the capability to capture allergens, cell debris and pathogens to facilitate their clearance from mucosal surfaces [6]. The functions of the MAMs include anti-adhesive, lubrication, water retention, pathogen barrier function [2, 7], anti-inflammatory effects [8, 9].

### Dry eye syndrome and mucins

Dry eye is a multifactorial disorder in the ocular surface, and causes ocular dryness, discomfort, visual disturbance and others. The disease mechanism include long-standing local inflammation in the conjuctiva and cornea, dysfunction of lacrimal gland with disturbance of tear secretion, meibomian gland dysfunction, reduction of mucin expression, and others, all of which potentially cause damage of the ocular surface epithelia [26, 27]. Although dryness of the ocular surface is a fundamental situation, local inflammation in the epithelium plays critical roles in the pathogenesis and symptom in patients with dry eye syndrome [26, 27]. Exaggerated evaporation of tear water increases the tear film osmolarity in dry eye. Besides the cellular response to the environmental hyperosmolarity through cation channel receptors, e. g. TRPV1, MAMs also could have the function of sensing the environmental hyperosmolarity through their extracellular domain and might transmit signal via their intracellular domain [28]. Expression of interleukin 6 (IL-6) and TNFα is upregulated by local tissue dryness in conjunctiva of dry eye patients and experimental dry eye model [29]. The desiccation of human corneal epithelial cell line cells increased IL-6 and TNFα level measured by ELISA [29]. In the dry eye model of rats, the IL-6 and TNFα mRNA level in the cornea measured by real-time RT-PCR increased [29]. It is quite interesting topic if mucin deficiency leads to inflammation in the ocular surface or vice versa, if upregulation of inflammatory cytokines/growth factors suppresses mucin expression in corneal and conjunctival epithelium. Expressions of MUC1, MUC2, MUC4, and MUC5AC are significantly lower in conjunctival epithelium gathered by impression cytology in the patients with dry eye syndrome compared with that in normal subject [30]. Especially, reduction of MUC1 expression in dry eye local tissue is reportedly most prominent and could be a marker for diagnosis or evaluation of the disease severity [30]. This point will be discussed in detail below. However, another study reports that expression of MUC1 protein and mRNA measured by western blotting and real-time PCR increases in the conjunctival epithelial cells gathered by impression cytology in the patients with Sjogren’s syndrome [31], suggesting further study is needed to uncover the mechanism of regulation of secretion of mucin components in dry eye-related ocular surface.

Topical benzalkonium chloride (BAC), a preservative in ophthalmic solution, induces reduction of tear film breakup time, increasing of corneal epithelial permeability, inflammatory infiltration, apoptosis, and squamous metaplasia. This condition could be investigated as an experimental model of dry eye in animals [32–34]. Topical BAC reduces MUC5AC-positive goblet cells in animal conjunctival fornix with impairment of arrangement of the microvillis of the corneal epithelium [32]. Immunolabeling revealed fewer MUC5AC-positive goblet cells in the BAC-treated conjunctival fornix in mice [32]. Cell culture study also showed the effects of an exposure to BAC in cultured corneal epithelial cells [35, 36]. mRNA expression of MUC1 and MUC16 measured by real-time RT-PCR was not changed, but protein level of a MUC1 and MUC16 examined by western blot analysis was reduced in cultured human corneal epithelial cells following exposure to eyedrops containing BAC solutions [35]. Currently most of the eyedrops contain preservatives, e. g. BAC, and thus, as stated above, it is required to consider effects of BAC on ocular surface epithelium and mucin condition.

### Anti-inflammatory roles of mucins

Mucins exhibit anti-inflammatory effects. Ocular surface epithelia are continuously exposed to allergens, debris, pathogens, desiccation, injury, and rubbing. Because mucins are considered to modulate local tissue inflammation as described above, it is hypothesized that the loss of a mucin member might induce expression of inflammatory cytokines in a mouse conjunctiva. Ocular surface mucins contribute differently to the protection of the ocular surface against allergens, pathogens, extracellular molecules, abrasive stress, and drying. Topical eyedrop of rebamipide, a drug that is capable of stimulating of mucin secretion in gastrointestinal tract or conjunctiva, is approved in Japanese government for use in the treatment of dry eye diseases (Table 2). It was reported that rebamipide exerts an anti-inflammatory effects on the ocular surface upon topical exposure to polyI:C in primary human conjunctival epithelial cells as revealed by quantitative RT-PCR analysis of mRNA expression of CXCL10, CXCL11, RANTES, MCP-1, and IL-6 [37].

**Table 2:** Application of clinical trial of eyedrop of rebamipide

• Dry eye • Sjogren’s Syndrome • Allergic conjunctivitis • Corneal epithelial disease	

### Allergic conjunctivitis and mucins

Impression cytology specimens from the patients with atopic keratoconjunctivitis picks up immunohistochemcal labeling for MUC5AC and MUC16 [38]. Expression of goblet cell-specific mucin, MUC5AC mRNA, is reduced and MUC16 mRNA expression is upregulated in brush cytology specimens from the patients with atopic keratoconjunctivitis [38]. On the other hand, patients with vernal keratoconjunctivitis have increased numbers of conjunctival goblet cells from impression cytology specimen [39]. Brush cytology specimens from eyes with atopic keratoconjunctivitis show higher MUC1, 2, and 4 and lower MUC5AC mRNA expression compared to the specimens from eyes with vernal keratoconjunctivitis [40], although the clinical manifestation of these two entities is quite similar. The reason for this discrepancy is to be uncovered. In mouse model of allergic conjunctivitis, repetitive application of allergens (cat danger or peptide P3-1) reduces the number of secretary granule-filled goblet cells and a decrease in Muc5AC and Muc4 mRNA measured by real-time RT-PCR [41]. After a period of 24 to 48 h, the number of goblet cells and mucin mRNA levels return to normal range [41]. Cytokines/growth factors expressed in the inflammatory/immune cells infiltrated to subconjunctival tissue are considered to be attributable to the alteration of mucins expression in the ocular surface. Modulation of mucin expression might potentially accelerate protective effects of tear mucous layer against external antigens that cause local allergic reaction.

### Phenotypes of a mutant mouse that lack each of MAMs

#### Muc1-null mouse

MUC 1 is reportedly involved in regulation of bacterial pathogen-related local tissue inflammation (Table 3) [42–45]. A Muc1-null mouse is fertile and grows overall normally [46]. However, lacking Muc1 leads to obvious phenotype in gastrointestinal tracts or airway epithelium [45, 47]. Muc1-null mice showed the increase of bacterial colonization of the stomach mucosa [43]. Muc1-deficient tissue of gastrointestinal tract or intestinal epithelial cell line with Muc1-silencing increase chemokine expression in response to exposure to TNFα in culture [43]. Muc1 play an important role in defense mechanism against external bacterial contamination. Muc1 knockout (KO) mice exhibited hyper- inflammatory response in the airways during acute experimental Pseudomonas aeruginosa- [44] or Haemophilis influenzae- lung infection [42] or in gastric mucosa upon Helicobacter infection [43]. Moreover Lacking Muc1 also impairs cholesterol uptake and absorption in the digestive tract [48]. In mice the loss of Muc1 significantly suppressed intestinal cholesterol absorption as compared with the wild-type mice, and the intestinal uptake rate of cholesterol is significantly decreased compared with the wild-type mice [48]. Muc1 is also involved in protective system against bacterial infection in airway epithelium [42, 44]. This mutant mouse does not exhibit a specific phenotype; the ocular surface of Muc1 null mice of C57BL/6 background appeared normal in all respects tested, namely, the structure of the ocular surface epithelia, as evaluated by using slit lamp biomicroscopy with fluorescein solution, bacterial adherence efficacy, and Muc4 mRNA expression in ocular surface [49]. Another paper reports that the incidence of conjunctivitis and blepharitis was higher in a Mun1-null mouse as compared with a wild type mouse [50].

**Table 3:** Characteristics of Muc knockout mice

Muc1 KO mice	Bacterial pathogen-related local tissue inflammation (gastrointestinal tract, airway)
Muc2 KO mice	Spontaneous colitis, colorectal canser
Muc5AC KO mice	Intestinal nematode infection
Muc13 KO mice	More severe dextran sodium sulfate (DSS)-induced colitis
Muc16 KO mice	Inflammmation (conjunctiva)

#### Muc2-null mouse

MUC2 is reportedly expressed in ocular surface [51, 52]. Although currently ocular phenotype of a Muc2-deficent mouse has not yet been well investigated, phenotype in other mucous organs, i.e., intestine, has well defined [8, 53] and serves as a reference in examination of ocular phenotype.

Expression of MUC2 is lowered in the patients with inflammatory bowel disease. MUC2 plays a significant role in the development of tissue inflammation, e. g., experimental colitis [8, 54, 55]. Muc2-deficient mice spontaneously developed colitis with diarrhea (Table 3), rectal prolapse, and failure to thrive presumably by commensal bacteria in tissue. Histological analysis of the colon of Muc2-null mice shows mucosal epithelial thickening, increased proliferation, and superficial mucous epithelial defect. The histologic damage in the colitis-inducing agent, dextran sulfate sodium (DSS)-treated Muc2-deficient mice are different compared with wild type and Muc2-heterozygous deficient mice, with many crypt abscesses [8]. In the absence of Muc2, the commensal bacteria are in direct contact with the epithelial cells and the bacteria invade into the tissue cells, causing infiltration of neutrophils and lymphocytes. Interestingly, aberrant intestinal crypt morphology and altered cell maturation and migration were recognized in Muc2-null mice [53]. Moreover, the mice develop colorectal cancer after several months (Table 3), further suggesting that the phenotype resembles the patients with ulcerative colitis [53].

#### Muc5Ac-deficient mouse lines

Wang et al. generated a transgenic mouse line with targeted deletion of goblet cells [56]. Goblet cells were deleted with expression of diphtheria toxin A driven by a human MUC5AC promoter. In this mouse model the external appearance of the ocular surface and corneal pathology was normal [56]. Muc4 expression compensated the decrease of Muc5AC by the loss of goblet cells as revealed by immunohistochemical staining, in situ hybridization, electronic microscopy, and RT-PCR [56]. Muc5AC protein is a critical component in the defense against intestinal nematode infection as revealed by using a Muc5AC deficient mouse model (Table 3) [57]. A related mouse line, a Muc5B-null mouse, also does not show ocular phenotype [58]. In another Muc5AC-null mouse line, corneal opacity was reportedly observed in 11 % of the animals, but others appeared to be normal [59]. In this mouse line there was a significant decrease of tear film break up time and increase fluorescein staining (epithelial disorder) on the ocular surfaces compared to WT mice [59]. Tear volume was determined with the phenol red thread tear test. Because total tear volume was not affected by the loss of Muc5AC, loss of Muc5AC might impair the quality of the tear fluid [59]. Further investigation is to be performed to uncover the roles of Muc5AC in ocular phenotype.

#### Muc13-null mouse

MUC13 is reportedly expressed in ocular surface [51, 52] and digestive organs [47, 60]. Muc13, a member of MAM, reportedly exhibits anti-inflammatory activity in mouse intestine [47, 60]. Lacking Muc13 causes spontaneous focal inflammation presumably by the action of commensal bacteria as well as potentiates dextran sodium sulfate-induced colitis in mice [60]. Muc13-null mice developed normally with no spontaneous intestinal pathology except mild focal neutrophilic inflammation in the small and large intestines of old mice [60]. However, they developed more severe acute colitis, as revealed by the loss of weight, rectal bleeding, diarrhea and histological colitis scores compared with wild-type mice when challenged with the colitis-inducing agent, dextran sodium sulfate (DSS) (Table 3) [60]. These findings promote us to hypothesize that gene ablation of Muc13 might induce an ocular phenotype. Similar to the situation in Muc2, ocular phenotype of a Muc13-deficent mouse is also to be investigated.

#### Muc16-null mouse

**Fig. 2:**
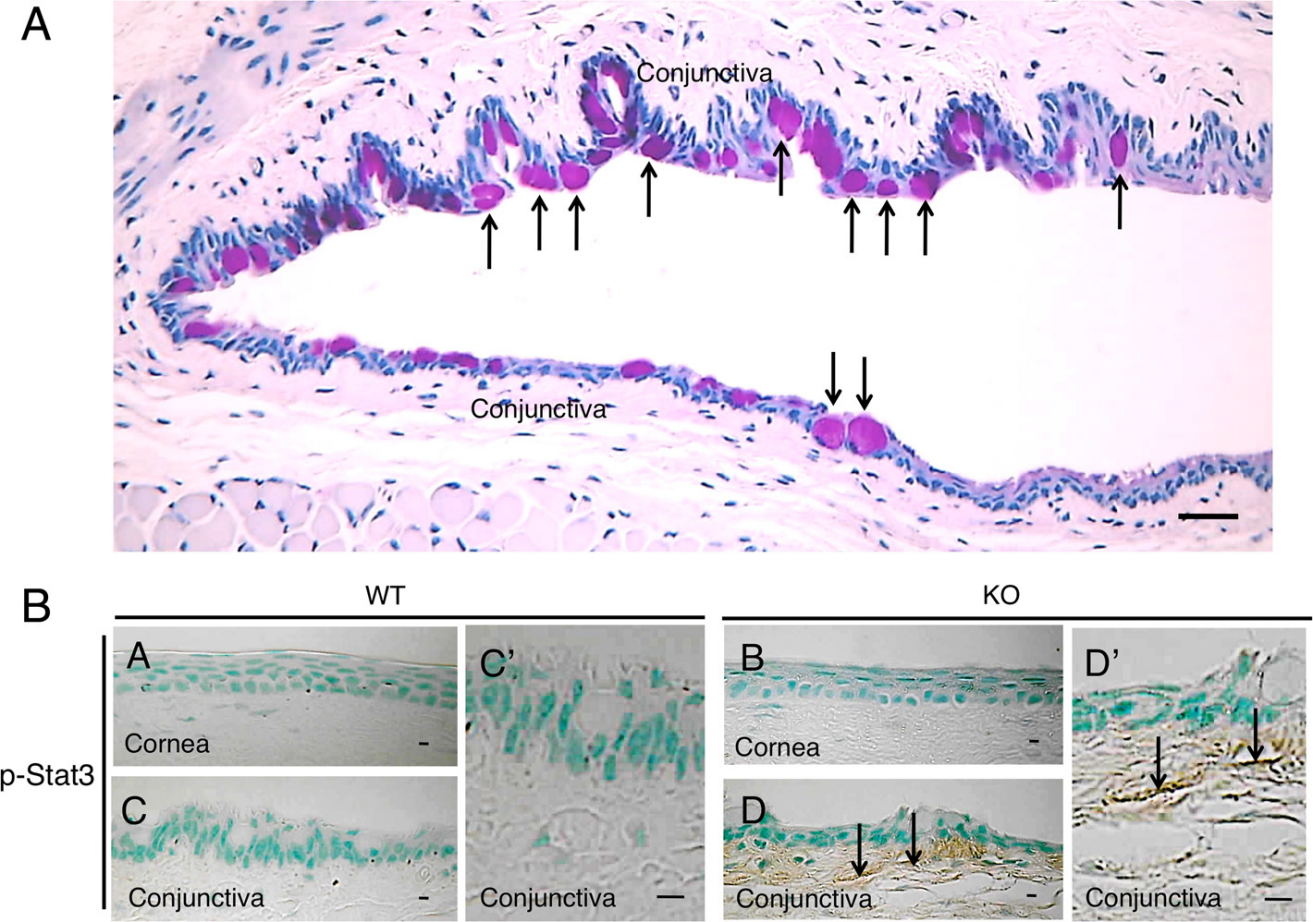
**a** Histology by Periodic acid-Schiff (PAS) staining of the conjunctival epithelium in WT mouse. In the conjunctiva, epithelial cells and subepithelial fibroblasts are seen. PAS positive goblet cells were distributed in the conjunctival epithelium (arrows). Bar, 50 µm. **b** Expression pattern of phsopho-Stat3 in ocular surface. Immunoreactivity for phospho-Stat3 was not observed in the corneal epithelium and keratocytes of both WT and KO mice as well as in the WT conjunctiva(A, B). However, phospho-Stat3 immunoreactivity was detected in fibroblasts (arrows) in the subepithelial connective tissue of KO mice (D, higher magnification in D’), but was not seen in the WT tissue (C, higher magnification in C’) Bar, 10 µm. Reproduced from Shirai et al. [9]

**Fig. 3:**
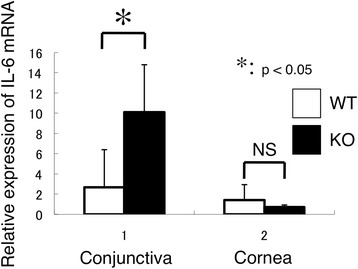
Expression of IL-6 in cornea and conjunctiva. The loss of Muc16 promotes the mRNA expression of IL-6 in the conjunctiva (*p < 0.05), but not affected the mRNA expression of IL-6 in the cornea. Reproduced from Shirai et al. [9]

**Fig. 4:**
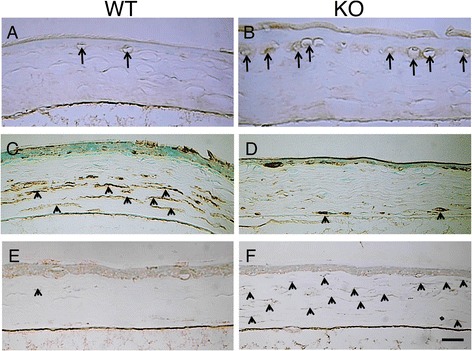
Immunolocalization of macrophages and analysis of keratocyte phenotype in healed cornea at 30 hours post-debridement. Immunohistochemistry detects F4/80-labeled macrophages, aldehyde dehydrogenase 3A1 (ALDH3A1, keratocyte marker), and -smooth muscle actin (SMA, myofibroblast marker). F4/80-labeled macrophages were more frequently observed beneath the regenerated epithelium in the KO mouse (**b**), while few F4/80-labeled cells were seen in the WT corneas (**a**). The cells in the posterior stroma were labeled for ALDH3A1, while fibroblastic cells in the anterior stroma were not labeled in the WT cornea (**c**). In the KO cornea, the majority of the cells in the stroma were negative for ALDH3A1 (**d**). A few -smooth muscle actin-positive myofibroblasts were detected in the WT stroma (**e**). Almost all the stromal cells were labeled with anti-SMA antibody and thus were myofibroblasts in the KO cornea (**f**). Bar, 50 μm. Reproduced from Shirai et al. [9]

Muc16 provides a protective barrier for the epithelial surface from bacterial adhesion and to suppress the immune system [25, 61–63]. Binding of fluorescently labeled Staphylococcus aureus to immortalized human corneal-limbal epithelial (HCLE) cells was measured to determine the role of MUC16 in the protection of pathogen adherence on the ocular surface epithelium [25]. An in vitro study sowed that knockdown of Muc16 gene expression in cultured human ocular surface epithelial cells promotes adhesion of Staphylococcus aureus on cell surface [25]. In vivo role in ocular surface epithelium was examined in Muc16-deficient mice, although Muc16 is expressed in conjunctival, but not in corneal epithelium, in mice, different from human [9]. Muc16-deficient homozygous mutant mice are fertile and appear normal without growth retardation [64]. Although the histology showed no obvious abnormalities in conjunctiva, subconjunctival fibroblasts express phospho-Stat3 with the absence of Muc16 (Fig. 2b) [9]. As described above, MAM members demonstrate anti-inflammatory activity. The loss of Muc16 exhibits inflammatory consequence in the conjunctiva (Table 3); it activates Stas3 signaling and upregulates expression of IL-6 (Fig. 1 and 3) [9]. IL-6 upregulation secondarily affects proliferation and migration of corneal epithelium [65, 66], as well as unfavorable fibrogenic process by keratocytes (by myofibroblast transformation) presumably via tear (Fig. 4) [9]. This is to be strongly noted that lacking this conjunctiva-specific mucin impairs the homeostasis of not only conjunctival but also corneal epithelium. The finding shows an important implication that dry eye-related conjunctival inflammation secondarily impairs homeostasis of the cornea.

### Conclusive remarks

In the future direction of research regarding mucins, analysis of ocular surface of mucin-gene related mutant mouse lines are to be further performed. Because drugs that stimulate mucin expression are clinically available. It is possible that we can suppose the decrease of the specific mucins in ocular surface disorder by the knowledge of the characteristics of the specific mucin deficient mice. Moreover, if the drugs that promote the expression of each mucin are developed, the increase of target mucin will lead to the treatment of ocular surface disease.
